# MetagenomicKG: a knowledge graph for metagenomic applications

**DOI:** 10.1093/bioinformatics/btag421

**Published:** 2026-06-23

**Authors:** Chunyu Ma, Shaopeng Liu, Stephanie Won, David Koslicki

**Affiliations:** Huck Institutes of the Life Sciences, Pennsylvania State University, State College, PA, United States; Insilicom LLC, Tallahassee, FL, United States; Department of Biology, Pennsylvania State University, State College, PA, United States; Huck Institutes of the Life Sciences, Pennsylvania State University, State College, PA, United States; Department of Biology, Pennsylvania State University, State College, PA, United States; Department of Computer Science and Engineering, Pennsylvania State University, State College, PA, United States; The One Health Microbiome Center, Huck Institutes of the Life Sciences, Pennsylvania State University, State College, PA, United States

## Abstract

**Motivation:**

The sheer volume and variety of genomic content within microbial communities makes metagenomics a field rich in biomedical knowledge. To traverse these complex communities and their vast unknowns, metagenomic studies often depend on distinct reference databases, such as the Genome Taxonomy Database (GTDB), the Kyoto Encyclopedia of Genes and Genomes (KEGG), and the Bacterial and Viral Bioinformatics Resource Center (BV-BRC), for various analytical purposes. These databases are crucial for the genetic and functional annotation of microbial communities. Nevertheless, the inconsistent nomenclature or identifiers of these databases present challenges for effective integration, representation, and utilization. Knowledge graphs (KGs) offer an appropriate solution by organizing biological entities from different databases to standardized identifiers, allowing their interrelations to be captured into a cohesive network regardless of the naming conventions used in each source. The graph structure not only facilitates the unveiling of hidden patterns but also enriches our biological understanding with deeper insights. Despite KGs having shown potential in various biomedical fields, their application in metagenomics remains underexplored.

**Results:**

We present MetagenomicKG, a novel knowledge graph specifically tailored for metagenomic analysis. MetagenomicKG integrates taxonomic, functional, and pathogenesis-related information on the human microbiome sourced from various databases, and further connects these with existing biomedical KGs to expand the biological network. Through various case studies involving the human microbiome, we demonstrate its utility in enabling hypothesis generation regarding the relationships between microbes and diseases, generating sample-specific graph embeddings, and providing robust pathogen prediction.

**Code Availability:**

The source code and technical details for constructing the MetagenomicKG and reproducing all analyses are available on GitHub at https://github.com/KoslickiLab/MetagenomicKG. The data used in this manuscript, including the pre-built files and use case input data, are archived on Zenodo with DOI: 10.5281/zenodo.17546861.

## 1 Introduction

Metagenomics, the study of microbial genetic material recovered directly from environmental samples, has become a powerful approach for characterizing microbial communities and their functional roles (Mendes *et al*. 2017, Ko *et al*. 2022, Shu and Huang 2022). This field offers profound insights into the composition and function of microbial communities and aids the discovery of novel genes, enzymes, and metabolic pathways applicable in medicine, biotechnology, and environmental science. The broad spectrum of microbial life also suggests a significant volume of unknowns, commonly referred to as “microbial dark matter” (Pedrós-Alió and Manrubia 2016, Bodor *et al*. 2020, Jiao *et al*. 2021). These unknowns encompass genetic material from unidentified and uncultured microbes, as well as their functions and interactions within microbial communities and environments. It is thus crucial for researchers to distinguish between what is genuinely novel and what has been previously observed or analyzed. This is particularly important in human-associated metagenomics, where microbial community composition and function are increasingly studied in relation to human health and disease. As a result, metagenomics relies extensively on reference databases to decipher microbial communities and advance scientific knowledge.

Access to well-established reference databases is crucial for metagenomic studies. Databases such as NCBI GenBank (Benson *et al*. 2013) and the Genome Taxonomy Database (GTDB) (Parks *et al*. 2022) offer a taxonomic framework for bacterial, archaeal and viral genomes, contributing to a deeper understanding of microbial phylogeny and taxonomy. The Kyoto Encyclopedia of Genes and Genomes (KEGG) (Kanehisa *et al*. 2017) provides a comprehensive source of information on genomes, biological pathways, diseases, and pharmaceuticals, which are crucial for functional annotation. The Bacterial and Viral Bioinformatics Resource Center (BV-BRC) (Olson *et al*. 2023) specializes in bacterial pathogens, offering a robust suite of data and tools for comparative genomic analysis to detect and characterize pathogens. Collectively, these and other databases form the backbone of metagenomic research, enabling annotation of genetic sequences, investigation of microbial functions, and analysis of the genetic basis of microbial communities. Furthermore, extensive knowledge about microbes and non-microbes is available in public databases (Consortium 2010, Lakin *et al*. 2017, Liu *et al*. 2022). However, despite their biological interrelatedness, these databases are typically accessed independently for specific purposes, making it challenging to fully leverage the wealth of information without effective integration.

Knowledge graphs (KG), data structures depicting real-world entities and their relationships, have become a key method for integrating databases and discovering new insights through their connections, particularly in structuring biomedical knowledge for translational science (Nicholson and Greene 2020, Fecho *et al*. 2022). KGs organize biological entities (e.g. genes, proteins, metabolic pathways, chemical compounds, and drugs) along with relationships between them into coherent, interconnected networks. By integrating existing knowledge databases derived from biological and biomedical data repositories, researchers can uncover hidden patterns and gain deeper insights into biological systems. For example, GenomicKB (Feng *et al*. 2023) consolidates existing knowledge on the human genome, epigenome, transcriptome, and 4D nucleome into a large KG for exploring specific patterns across omics data. RTX-KG2 (Wood *et al*. 2022) combines data from 70 biomedical knowledge bases to construct a KG on which powerful computational reasoning resources were developed for biomedicine (Ma *et al.* 2023). KG-COVID-19 (Reese *et al*. 2021) integrates COVID-19 data sources to expedite the discovery of new treatments. These examples demonstrate the value of KGs for large-scale biomedical knowledge integration and research applications. However, efforts to apply KGs to metagenomics remain limited. Currently, there is no dedicated KG specifically for metagenomic research. Joachimiak *et al*. (2021) built KG-Microbe using Natural Language Processing (NLP) techniques but excluded any biomedical information (e.g. diseases). Santangelo *et al.* constructed MGMLink (Santangelo *et al.* 2025) via gutMGene (Cheng *et al*. 2022) and PheKnowLator framework (Callahan *et al.* 2024) to assess mechanistic hypotheses linking gut microbes to diseases. However, its utility in metagenomics research is limited by its reliance on a small number of gutMGene-described microbes and the absence of an efficient method for mapping newly identified microbes to the KG. Goetz *et al.* created MicrobiomeKG (Goetz *et al*. 2025), a literature-derived knowledge graph of microbe-to-host health associations built by mining supplementary tables from published microbiome studies and applying a neural-network edge score to heterogeneous evidence for hypothesis generation. Various microbiome related knowledge graphs offer opportunities to investigate host-microbial relationships, however, most of them lack breadth or depth of curated biomedical information. To date, no KG integrating metagenomic taxonomic resources with biomedical knowledge of human disease exists.

To address these limitations in human-associated metagenomics, we developed MetagenomicKG, a knowledge graph combining metagenomic data with biomedical knowledge to explore microbe-disease relationships. It integrates widely used taxonomic information (e.g. GTDB and NCBI taxonomies) with key biomedical knowledge resources, such as KEGG, BV-BRC, and RTX-KG2. These sources encompass biological functions, systems, pathogenic microbes, phenotypic features, and diseases. MetagenomicKG is designed to enhance metagenomic data mining capabilities and uncover the underlying relationships between microbes and diseases. To demonstrate its utility, we present several use cases including hypothesis generation, microbe–disease exploration, sample-specific graph-based embeddings, and pathogen prediction.

We envision MetagenomicKG as a comprehensive multi-modal reference framework for investigating the role of microbial communities in human disease. It is designed to support a range of exploratory analyses of microbe-human disease associations. Additionally, it can serve as a structured knowledge repository for machine learning applications, facilitating the development of predictive models and analytical tools. Given the extensible nature of KGs, MetagenomicKG will continue to evolve through the integration of additional databases, thereby expanding its utility and driving new discoveries in microbial-disease research.

## 2 Graph overview

**Figure 1:**
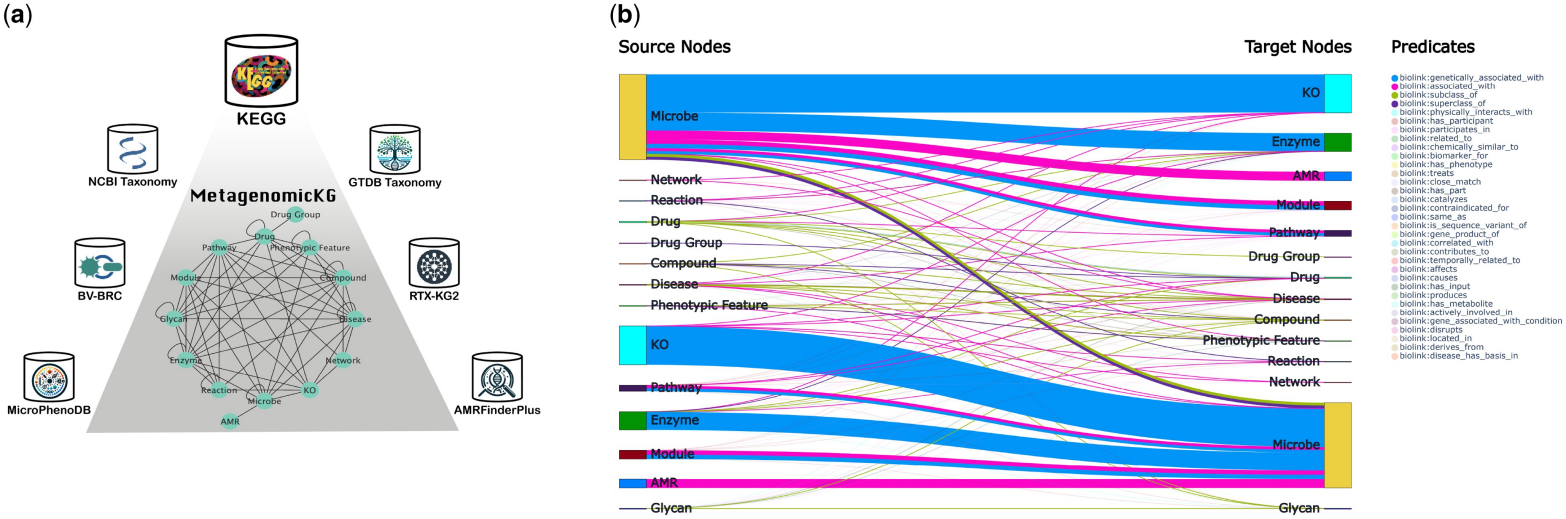
Architecture of MetagenomicKG. (a) Schematic representation showing the meta-knowledge graph (grey triangle) integrating seven heterogeneous biomedical databases. (b) Sankey diagram displaying edge predicate distribution between node types. Colored rectangles represent node types; colored flow lines represent Biolink predicate types. Line thickness is proportional to edge predicate frequency between node types. The top 4 most frequent predicates are shown with full opacity; remaining predicates at 25% transparency.

Our MetagenomicKG integrates data from seven sources: GTDB taxonomy (Parks *et al*. 2022), NCBI taxonomy (Schoch *et al*. 2020), KEGG (Kanehisa *et al*. 2017), RTX-KG2 (Wood *et al*. 2022), BV-BRC (Olson *et al*. 2023), MicroPhenoDB (Yao *et al*. 2020), and NCBI AMRFinderPlus Prediction (Feldgarden *et al*. 2021) (see Fig. 1(a)). MetagenomicKG is a directed multigraph comprising 1.25 million nodes and 56 million edges. Nodes are categorized into 14 distinct types, largely reflecting KEGG database classifications: Microbe, Phenotypic Feature, Disease, KEGG Orthology (KO), Compound, Reaction, Drug, Glycan, Enzyme, Drug Group, Antimicrobial Resistance (AMR), Network, Pathway, and Module. To support data comparison and interoperability, we adhere to the semantic schemas of established biomedical KGs (Bizon *et al*. 2019, Reese *et al*. 2021, Wood *et al*. 2022, Morris *et al*. 2023), mapping node and edge types to the standardized categories and predicates defined by the Biolink model (Unni *et al*. 2022). The Biolink model is an open-source framework that standardizes biological and translational science data to support interoperability across KGs. Table 1 shows all node types in MetagenomicKG with their mapping to the Biolink entity category and corresponding explanations. Figure 1(b) illustrates all Biolink edge predicates used in the graph, along with their distribution across different node types. Currently, the top four predicates with the most edges in MetagenomicKG are *biolink: genetically_associated_with*, *biolink: associated_with*, *biolink: subclass_of*, *biolink: superclass_of*. These predicates are predominantly associated with the node types Microbe, KEGG Orthology (KO), Enzyme, Module, and Pathway.

**Table 1:** Description of node types used in MetagenomicKG.

Node Type	Biolink Mapping	Description
Microbe	biolink: OrganismTaxon	An individual (e.g. strain) or a set of organisms (taxonomy rank higher than strain such as species) that belong(s) to either of bacteria, archaea, viruses, fungi from NCBI taxonomy or GTDB taxonomy or both.
Phenotypic Feature	biolink: PhenotypicFeature	Any observable characteristic of an individual that results from the interaction between its genotype and the environment, such as nasal dryness, collected from RTX-KG2.
Disease	biolink: Disease	A disorder that affects the structure or function of the body, resulting in specific signs, symptoms, or phenotypes, from either KEGG, RTX-KG2, BVBRC or MicroPhenoDB.
KEGG Orthology	biolink: BiologicalEntity	An orthologous group defined by KEGG that contains a group of genes or proteins representing specific gene functions or sets of related functions.
Compound	biolink: MolecularEntity	An entity representing small molecules, biopolymers, and other chemical substances that are relevant to biological systems, mostly from KEGG and RTX-KG2.
Reaction	biolink: MolecularActivity	A chemical reaction, mostly enzymatic reaction, defined by KEGG.
Drug	biolink: Drug	A substance intended for use in the diagnosis, cure, mitigation, treatment, or prevention of disease, mostly from KEGG and RTX-KG2.
Glycan	biolink: MacromolecularComplex	A complex carbohydrate involved in various biological processes from KEGG.
Enzyme	biolink: Polypeptide	A biological catalyst that accelerates a chemical reaction in a living organism, mostly from KEGG.
Drug Group	biolink: MolecularMixture	A group of drugs defined by KEGG that are organized based on their chemical structure, similarity in structure, shared drug targets, common mechanisms of action, or interactions with drug metabolizing enzymes and transporters.
Antimicrobial Resistance	biolink: Protein	An antimicrobial resistance (AMR) gene identified by the AMRFinderPlus tool.
Network	biolink: NamedThing	A molecular interaction and reaction network defined by KEGG that is used to study diseases and drug mechanisms.
Pathway	biolink: Pathway	An entity about molecular pathway for metabolism, genetic and environmental information processing, cellular processes, organismal systems, human diseases, and drug development.
Module	biolink: BiologicalProcess	A functional unit of genes and reactions organized to represent specific metabolic pathways or phenotypic features by KEGG.

### 2.1 Data sources

Here, we detail the seven data sources currently integrated into MetagenomicKG, grouped into four categories. Each category is designed to be extensible, allowing the incorporation of new data sources as they become available. This ensures that MetagenomicKG evolves with the expanding landscape of metagenomic research. Additionally, we highlight several critical tools used to establish edges (connections) between biological entities, further enhancing the graph’s utility and interconnectivity.

#### 2.1.1 Taxonomy

The GTDB and NCBI databases are two prominent taxonomic frameworks in metagenomics, each with unique advantages and limitations. The NCBI taxonomy database is highly comprehensive, providing taxonomic annotations for nearly all life forms, including bacteria, archaea, viruses, and eukaryotes. However, it lacks phylogenetic accuracy in metagenomic studies (Wirth and Bush 2023). In contrast, GTDB is limited to bacteria and archaea but offers a standardized, genome-based classification system that is particularly valued by metagenomics researchers (Parks *et al*. 2022). To balance taxonomic accuracy with broad coverage, we predominantly utilize the GTDB taxonomy, incorporating bacterial and archaeal genomes from NCBI RefSeq and BV-BRC into the GTDB framework using its genome annotation tool, GTDB-Tk. For microbial types absent from GTDB (e.g. viruses and fungi), we rely on taxonomic information from NCBI.

##### 2.1.1.1 NCBI taxonomy

– The largest primary taxonomic system for the classification and nomenclature of all sequenced organisms represented in the International Nucleotide Sequence Database Collaboration’s nucleotide and protein records. It provides a comprehensive framework for the up-to-date classification and naming of viruses and fungi. We only use its viral and fungal taxonomic information (downloaded on 30 August 2023) in MetagenomicKG.

##### 2.1.1.2 GTDB taxonomy

– A standardized, genome-based taxonomic database for bacteria and archaea built on a comprehensive phylogenetic framework. It leverages large-scale, high-quality genomic data to establish a consistent and evolutionarily informed taxonomy, enabling a more accurate representation of microbial diversity and evolutionary relationships. GTDB has been widely adopted in metagenomics research (Nissen *et al*. 2021, Smith *et al*. 2022, Blanco-Míguez *et al*. 2023). We integrate its latest version (Release 214) into MetagenomicKG.

#### 2.1.2 Functional annotation

In addition to taxonomic classification, we incorporated functional annotation data to elucidate the roles microbes play within their ecosystems. Functional context is essential for accurately interpreting microbial activity beyond taxonomy. Therefore, KEGG was the most suitable resource for this purpose (Aramaki *et al*. 2020). MetagenomicKG is built using KEGG as its foundation, aligning with the data types defined within KEGG databases.

##### 2.1.2.1 KEGG

– A key database that provides comprehensive molecular and functional annotations based on genome sequences and other high-throughput data. It offers a wide range of resources, such as pathways, compounds, glycans, reactions, human diseases, drugs, drug groups, functional orthologs, and their interconnections. The content of MetagenomicKG is primarily based on data downloaded from the KEGG FTP server on 11 October 2023.

#### 2.1.3 Pathogen characterization

To apply MetagenomicKG to pathogen prediction, we include BV-BRC and MicroPhenoDB, data resources related to known pathogens, and use them to label microbial entities as pathogens and add their connections to other biological entities.

##### 2.1.3.1 BV-BRC

– An advanced research platform formed by integrating the PATRIC (Gillespie *et al*. 2011), IRD (Zhang *et al*. 2017), and ViPR (Pickett *et al*. 2012) databases. BV-BRC provides extensive data on bacterial and viral pathogens. All bacterial and viral genomes, along with their metadata, were downloaded via the BV-BRC FTP server on 7 June 2023. Only genomes with ‘human’ as the host and a clearly associated disease in the metadata were classified as pathogens and included in MetagenomicKG.

##### 2.1.3.2 MicroPhenoDB

– A database that maps the relationships between pathogenic microbes, their core genes, and human disease phenotypes. It was developed through manual literature curation with a scoring framework that integrates evidence from the Infectious Diseases Society of America (IDSA) guidelines and other manually curated sources. Data from this database were downloaded on 30 May 2023.

#### 2.1.4 Existing biomedical knowledge graphs

To enrich MetagenomicKG with diseases and their associated phenotypic features, we integrate extensive connections from RTX-KG2 (Wood *et al*. 2022) into MetagenomicKG.

##### 2.1.4.1 RTX-KG2

– One of the largest open-source, biomedical knowledge graphs built based on the Biolink model. It contains extensive knowledge from 70 databases, including human-curated sources, computationally derived data, and information from scholarly publications. The RTX-KG2 (version 2.8.4) used in MetagenomicKG contains approximately 6.8 million nodes and 45.36 million edges. Since RTX-KG2 includes many node types unrelated to metagenomics (e.g. “biolink: GeographicLocation” and “biolink: Device”), we extracted only knowledge relevant to microbe-disease relationships, specifically nodes of types ‘biolink: Disease’ and ‘biolink: PhenotypicFeature,’ along with their adjacent edges. Additionally, we excluded the RTX-KG2 edges supported solely by the Semantic MEDLINE Database (SemMedDB) due to known issues with contradictory or erroneous relationships (Cong *et al*. 2018) caused by the SemRep algorithm (Rindflesch and Fiszman 2003).

#### 2.1.5 Bioinformatics tools

To facilitate MetagenomicKG in incorporating new or unidentified genomes, we used three tools from different platforms to obtain annotation information and integrated it with existing knowledge in the graph.

##### 2.1.5.1 GTDB-tk

– A taxonomic classification tool developed by the Genome Taxonomy Database (GTDB) team to provide standardized and objective taxonomic assignments for bacterial and archaeal genomes. It leverages a marker-gene-based reference tree, using HMMER for gene alignment and pplacer for genomic placement within the reference tree. Classification is then determined based on Relative Evolutionary Divergence (RED), Average Nucleotide Identity (ANI), and the established phylogenetic positioning. We utilized GTDB-tk to harmonize genome FASTA files from various databases, ensuring accurate integration of each new genome into the appropriate node within our graph.

##### 2.1.5.2 KofamKOALA

– A tool designed by the Kyoto Encyclopedia of Genes and Genomes (KEGG) for the functional annotation of genome sequences. It utilizes a database of KEGG Orthology (KO) terms linked to specific metabolic pathways and functions, offering a precise interpretation of protein roles in the cellular context. The tool uses HMMER-based searches to align input sequences against a set of curated reference profiles corresponding to the KO terms, ensuring high-confidence prediction of protein functions. We used KofamKOALA to annotate genomic sequences to establish connections to the functional layer in our graph.

##### 2.1.5.3 NCBI AMRFinderPlus prediction

– A tool developed by the National Center for Biotechnology Information (NCBI) to identify antimicrobial resistance (AMR) genes, resistance-associated point mutations, and other related genetic elements in bacterial genomes. It utilizes the BLASTX protein search against the NCBI Bacterial Antimicrobial Resistance Reference Gene Database for AMR protein identification. We applied this tool to the genome sequences of the databases mentioned above to predict their potential AMR genes.

### 2.2 Construction

The MetagenomicKG build process is automated via a parallel workflow framework, Snakemake, which uses both Python and shell scripting. The definition of node categories in MetagenomicKG primarily adheres to the database types used in KEGG, with additional categories for Microbe, Phenotypic Feature, and Antimicrobial Resistance. The edge categories are aligned with the definitions used in the Biolink Model (version 3.5.2). The graph is constructed sequentially by integrating data from GTDB Taxonomy, NCBI Taxonomy, KEGG, RTX-KG2, BV-BRC, MicroPhenoDB, and AMRFinderPlus. Each biological entity identifier from these data sources is mapped to one of the node types described in Table 1 and is assigned a unique new node ID composed of the node type and an index number (e.g. 1, 2, …). The bacterial and archaeal genomes from each data source are assigned to the closest GTDB identifier via GTDB-Tk (Chaumeil *et al*. 2020) if they lack RefSeq or GenBank assembly identifiers, based on an Average Nucleotide Identity (ANI) threshold of ≥ 99.5%. Otherwise, other microbial genomes are unified by mapping their RefSeq and GenBank assembly identifiers. To resolve inconsistent nomenclature or identifiers, different identifiers and names of the same biological entity are conflated via the Unified Medical Language System (UMLS) search function (UMLS Search API: https://documentation.uts.nlm.nih.gov/rest/search/), Ontology mapping with Ontology Xref Service (OxO) (OxO REST API: https://www.ebi.ac.uk/spot/oxo/docs/api), as well as Node Synonymizer function provided by RTX-KG2 (Wood *et al*. 2022). The information of all nodes and edges in the MetagenomicKG is stored in two separate files with a tabular format and imported into the Neo4j graph database for Cypher querying. Each node in the graph contains the following information: node ID, node type, all possible names (“all_names” column), description, original data source (“knowledge_source” column), source links, synonyms, pathogen label (“is_pathogen” column). Each edge includes the source node, target node, predicate description, and original data source (“knowledge source” column), where both source and target nodes use the new node ID consistent with the one used in the node table. To ensure the reliability of nodes and edges, the “knowledge source” and “links” attributes can be used to trace them back to their original data sources.

## 3 Applications of metagenomics knowledge graph

In this section, we demonstrate the versatility of MetagenomicKG through several use cases that highlight its potential applications in metagenomics research.

### 3.1 Metagenomic resource integration for hypothesis generation and exploration

Investigating the relationships between pathogens, their molecular pathways, and associated diseases requires researchers to query multiple databases with no efficient way to traverse connections across taxonomy, function, and disease. MetagenomicKG addresses this by integrating these resources into a single queryable framework with reduced redundancy, allowing more efficient query and exploration of cross-database biological relationships that would otherwise require significant manual effort to assemble. For example, although the BV-BRC database records over 15 000 human pathogens, many are highly similar, sharing more than 99.5% ANI with other genomes in the collection. To efficiently streamline taxonomic representation, we consolidated these genomes, along with pathogens from KEGG, into 2682 unique strain-level nodes linked to 405 species from GTDB. This aggregation enables an efficient, scalable taxonomic structure and supports user-friendly exploration of biological relationships among pathogenic genomes.

To associate microbial genomes with diseases through molecular functions and pathways, MetagenomicKG links genomes and their higher-order taxonomic groups to KEGG functional layers, which in turn connect to other KEGG databases, revealing functional mechanisms that underlie pathogenicity. Although not all microbial genomes are represented in KEGG, shared proteins—such as those associated with Antimicrobial Resistance (AMR) genes and Virulence Factors (VF)—can bridge genomes with unknown molecular mechanisms to those with known mechanisms. By integrating MetagenomicKG with RTX-KG2, we extend its scope beyond the microbiome to encompass broader disease context and phenotypic features.

**Figure 2:**
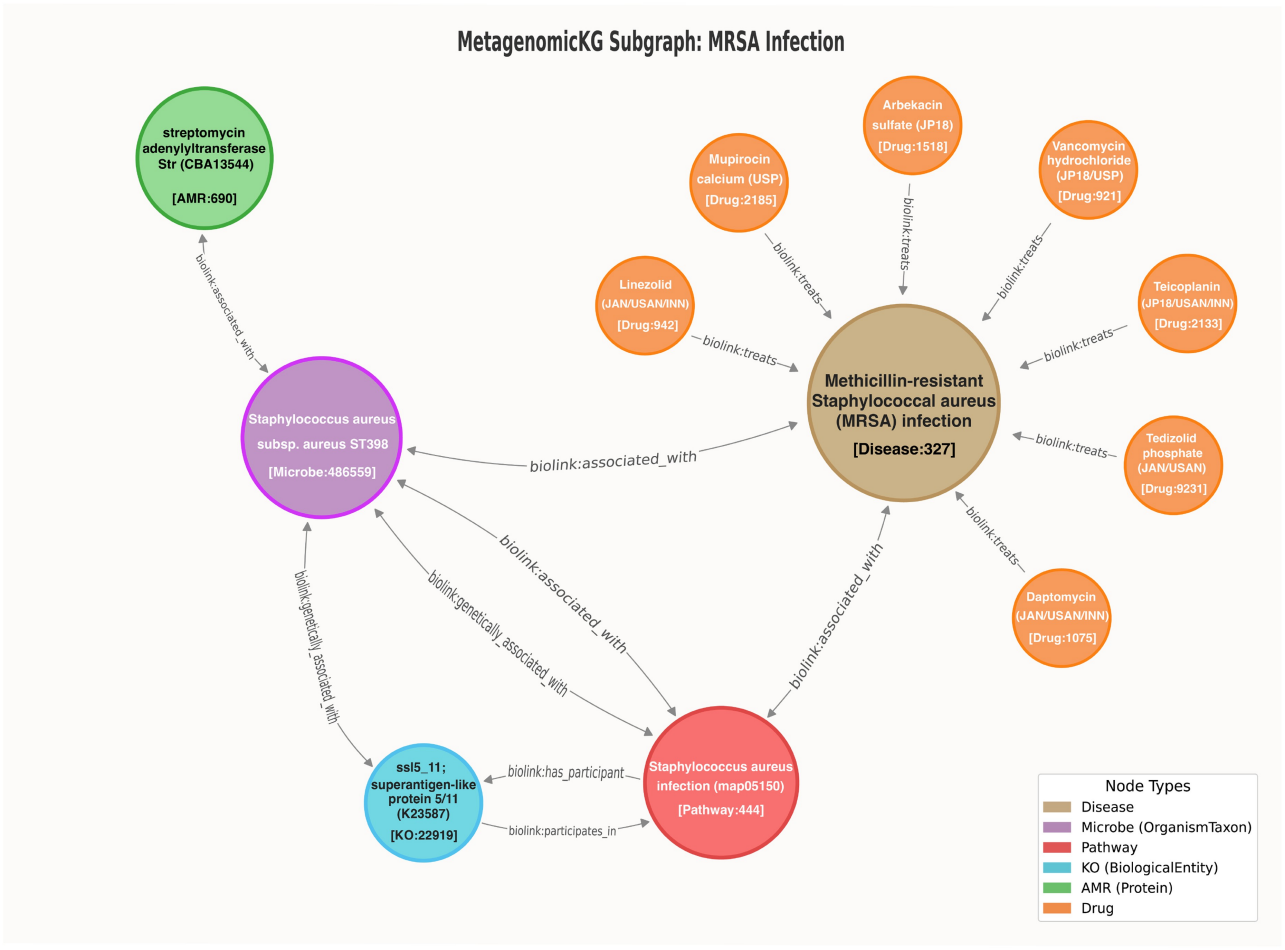
A subgraph representing the MRSA disease, its treatment, its causative pathogen, and relevant functional unit and pathway, as well as the known AMR protein. Each node displays its name and MetagenomicKG node ID (in brackets). The subgraph was generated using the query provided in Supplementary Material Section S2, available as supplementary data at *Bioinformatics* online, Usecase 1.

The MetagenomicKG integration system provides a holistic approach to investigating the relationships between pathogens, their functions, and the discovery of potential drug targets. For example, Staphylococcus bacteria are usually harmless but can cause serious infections that are resistant to treatment due to antibiotic resistance (Centers for Disease Control and Prevention: Methicillin-resistant Staphylococcus aureus (MRSA)). Figure 2 illustrates this case with a subgraph resulting from a query (See Supplementary section S2, available as supplementary data at *Bioinformatics* online for details.) on the graph showing the associations among AMR: streptomycin resistance protein (green node), Microbe: Staphylococcus *aureus* subsp. *aureus* ST398 (purple node), KO: K23587 (blue node), Pathway: map05150 (red node), Disease: Methicillin-resistant Staphylococcal aureus (MRSA) infection (brown node) and Drugs (orange node). This subgraph indicates that a particular strain of Staphylococcus, Staphylococcus *aureus* subsp. *aureus* ST398, is capable of causing MRSA infections via mechanisms involving KO functional unit of superantigen-like protein 5/11 and KO pathway of map05150. The pathogen carries the AMR protein CBA13544, which can be targeted by several drugs for treatment. This example demonstrates MetagenomicKG ’s capacity to support metagenomic data exploration and can be extended to more complex analyses. For instance, another genome with similar characteristics, possessing key genes in the same pathway and AMR genes that counteract relevant drugs, could likewise be flagged as a potential pathogen.

### 3.2 Generation of sample-specific graph embeddings

While taxonomic and functional profiling are commonly used to characterize metagenomic communities, they often fail to reveal the underlying biological relationships among microbes, metabolic pathways, and diseases. MetagenomicKG, combined with graph-based techniques, presents a novel approach for exploratory data analysis of metagenomic samples by leveraging graph embeddings to integrate diverse metagenomic reference knowledge. To better incorporate the existing biological knowledge into the representation of a metagenomic sample, we generated a sample-specific embedding (i.e. a numerical vector representation) using a modified version of the PageRank algorithm (Page *et al*. 1999), which effectively integrates knowledge from graphs and external data sources (Nelson *et al*. 2019). This approach enables the combination of taxonomic or functional profiling results with metagenomic knowledge in MetagenomicKG to produce sample-specific graph embeddings. The length of each embedding equals the number of nodes in MetagenomicKG, and each value indicates the relative importance of the corresponding node for a given sample. These embeddings support a variety of downstream analyses, including differential analysis of important sample-related biological entities, disease status classification (Huang *et al*. 2017, Luo and Long 2020), and network analysis to elucidate microbial community structures (Liu *et al*. 2021). They facilitate the discovery of hidden patterns and connections that conventional analysis may overlook.

**Figure 3:**
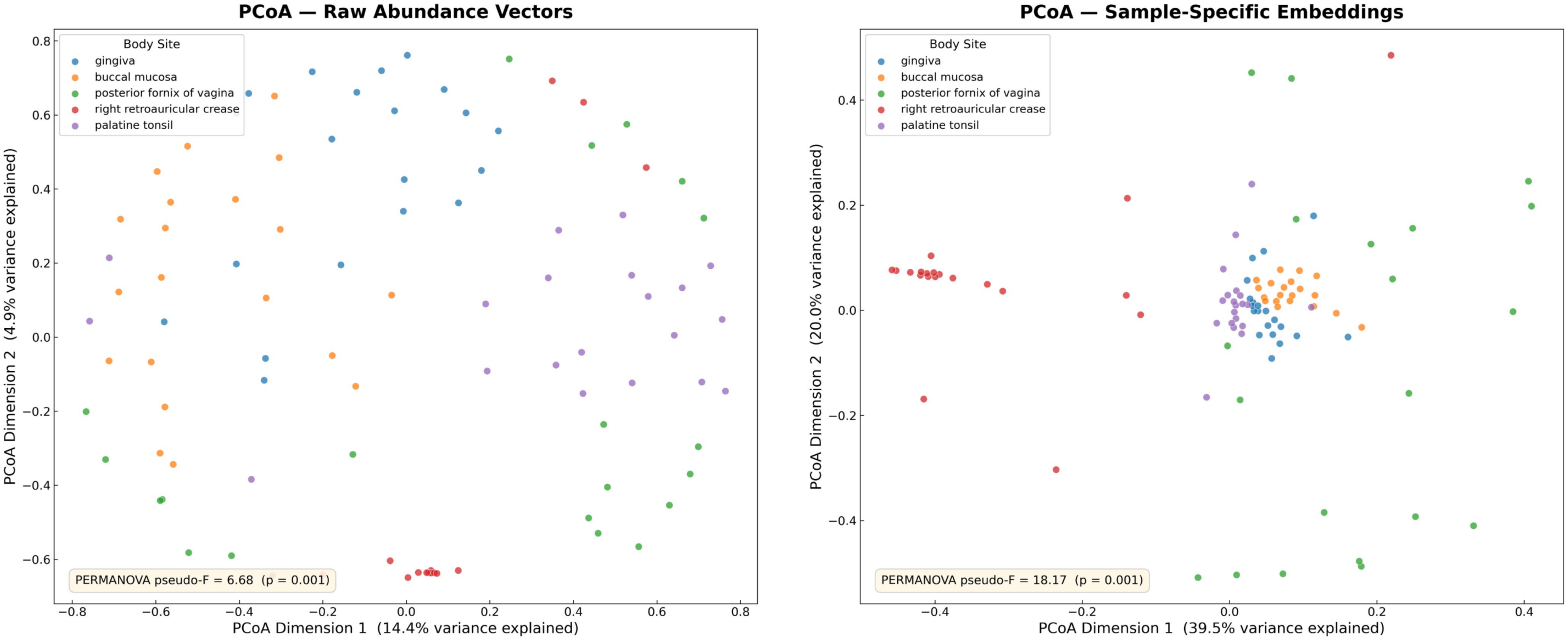
Comparison of PCoA visualizations for 100 randomly selected HMP samples using (left) raw abundance vectors generated by Sourmash and (right) sample-specific embeddings generated by the personalized PageRank algorithm. Axis labels show the variance explained by the first two PCoA dimensions, and each panel includes the PERMANOVA pseudo-F statistic (999 permutations) computed from the full distance matrix.

To demonstrate this approach, we used real data from the Human Microbiome Project (HMP) and BV-BRC databases. Taxonomic profiles and sample-specific graph-based embeddings were generated for 100 randomly selected samples from five body sites to perform clustering analysis. The Principal Coordinates Analysis (PCoA) results are presented in Fig. 3 (See more method details in Section S2, available as supplementary data at *Bioinformatics* online in the supplementary material). To distinguish true biological separation from possible projection artifacts, we quantified both the variance explained by the first two PCoA axes and the PERMANOVA pseudo-F statistic (Anderson 2001) computed from the full distance matrix. For the raw abundance representation, the first two PCoA axes explain 19.3% of the total variance, whereas for the sample-specific embeddings they explain 59.5%, indicating that the embedding-based representation is much more faithfully preserved in two dimensions. Likewise, PERMANOVA showed substantially stronger body-site segregation for the embeddings than for the raw abundance data (pseudo-F = 18.17 vs. 6.68); *P* = 0.001 for both).

The results support our expectation that samples from distinct body sites exhibit distinct microbial compositions, while closely related sites (e.g. palatine tonsil, buccal mucosa, gingiva) show greater similarity. Both taxonomic and graph-based approaches yield clear cluster boundaries, but the graph-embedding-based method captures deeper biological patterns, drawing samples from closely related body sites into closer proximity. This suggests that graph embeddings in metagenomic studies can help prioritize biologically meaningful features beyond conventional taxonomic or functional profiles. Our demonstration highlights the effectiveness and potential utility of MetagenomicKG, leveraging graph embeddings to drive deeper, knowledge-informed metagenomic analysis.

### 3.3 Pathogen predictions

Detecting pathogenic organisms is crucial for clinical diagnostics, public health risk prevention, and understanding environmental microbiomes (Yang *et al*. 2020, Ko *et al*. 2022, Wang *et al*. 2022). Traditional amplicon-based methods for pathogen detection start with conserved gene families that are commonly shared among strains in the same species, such as 16S rRNA for bacteria and 18S rRNA for protozoa and fungi. These methods utilize deep amplicon sequencing (DAS) technique to amplify and detect taxonomic markers in pathogenic species (Miller *et al*. 2013). But these DAS-based methods require prior knowledge of potential pathogenic agents, leading to the omission of pathogenic organisms with unknown markers. Unlike the DAS technique, shotgun sequencing captures all nucleic acids present in a sample, allowing for the detection of rare and unknown pathogens. Sequence-based models, such as alignment-based methods (Altschul *et al*. 1990) and k-mer-based methods (Rosen *et al*. 2011, Wood and Salzberg 2014), have been popular in recent years. Machine learning methods have also evolved to predict pathogen genomes by learning sequence patterns or alignment status (Deneke *et al*. 2017, Bartoszewicz *et al*. 2020, 2021, Jiang *et al*. 2023). However, to the best of our knowledge, all existing machine learning approaches (e.g. PaPrBaG (Deneke *et al*. 2017), DeePac (Bartoszewicz *et al*. 2020)) rely on the sequence-based features from known pathogens for prediction. Such homology-based approaches can struggle to identify novel or distantly related pathogens. This creates a major bottleneck for detecting unknown pathogens, especially when rare or no closely related reference genomes exist in the training data. The MetagenomicKG can address this drawback by leveraging functional connections, taxonomic relationships, and additional biomedical connections to predict potential pathogenicity. Novel pathogens, which share limited sequence similarities with known reference databases, can still be detected through the graph structure which captures additional information about pathways, proteins, biological processes, etc.

To demonstrate the advantage of MetagenomicKG in pathogen identification, we applied a simple graph neural network (GNN) to MetagenomicKG (see more details in section S3 in the supplementary material, available as supplementary data at *Bioinformatics* online) and compared its performance with other deep learning, sequencing-based models in binary pathogen classification. For ground-truth data, we curated a list of reliable human-associated pathogens from KEGG and BV-BRC (see more details in section S3, available as supplementary data at *Bioinformatics* online).

**Figure 4:**
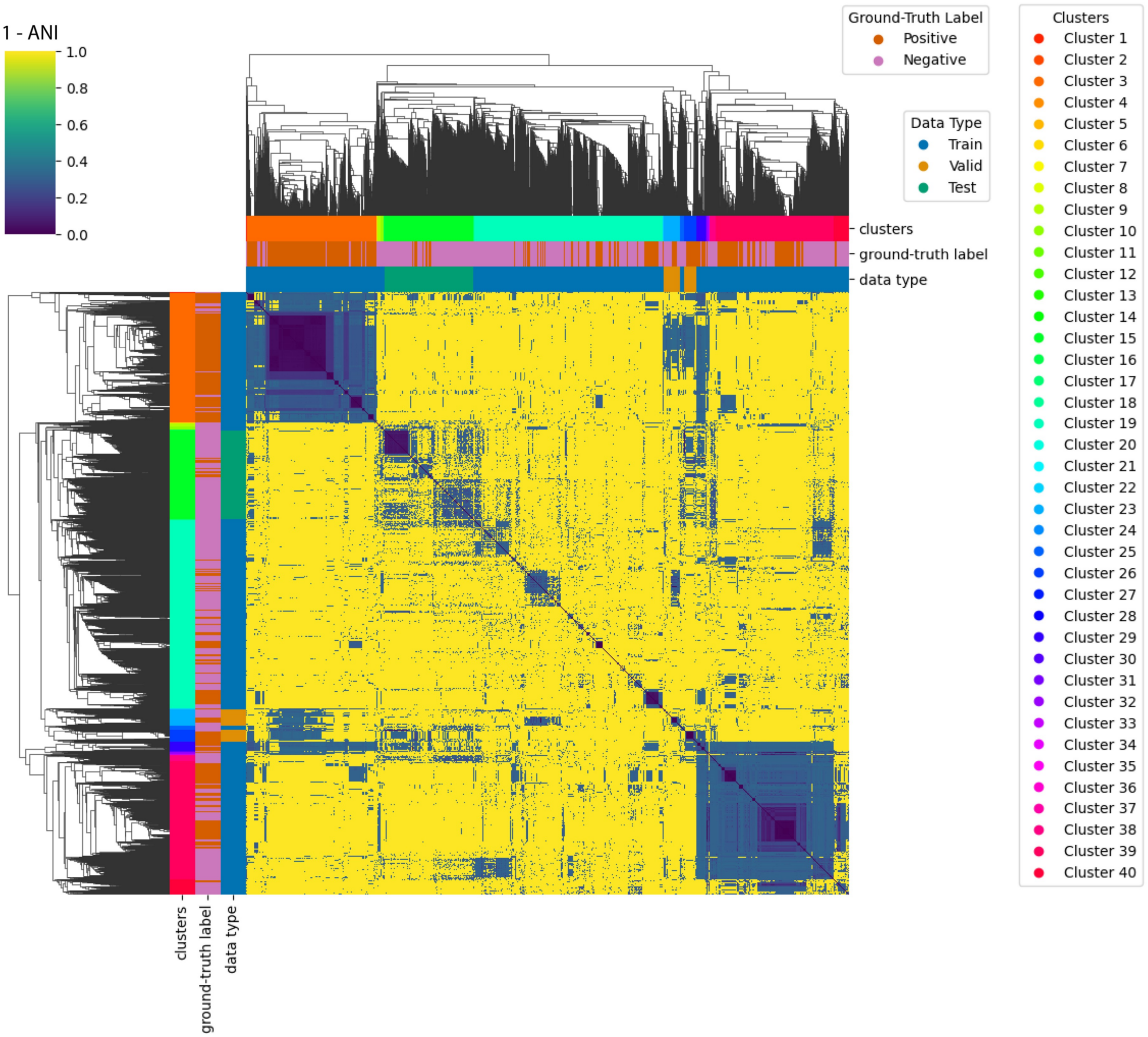
Heatmap plot with hierarchical clustering in two dimensions to show the sequence similarity of ground-truth genomes used in the “pathogen predictions” use case. The genomes are clustered into 40 groups based on 1 - ANI as distance. The three color bars correspond to ‘clusters’, ’ground-truth label’, and ‘data type’ classifications, respectively. The ‘positive’ genomes are the ground-truth pathogenic microbes while the ‘negative’ genomes are the ground-truth non-pathogenic microbes.

To present MetagenomicKG ’s superiority in identifying pathogens lacking sequences similar to those in the training dataset, we strategically divided the ground-truth data into training, validation, and test sets using clustering based on average nucleotide identity (ANI). This approach, which we refer to as the “Missing Reference Split,” ensures that genomes in the test set are not closely related to those in the training set. We first used Sourmash (Pierce *et al*. 2019) to compute pairwise ANI scores for all ground-truth genomes and then clustered them based on the score. Figure 4 shows the hierarchical clustering of all ground-truth genomes in two dimensions using 1 - ANI as the distance metric. The clustering reveals that many pathogenic genomes group tightly, indicating high sequence similarity. To simulate scenarios where reference genomes for unknown pathogens are unavailable, we intentionally selected test samples from clusters with low similarity to the training set (e.g. cluster 15). Table 2 presents the distribution of ground-truth data across training, validation, and test sets. For comparison, we also trained models using a 10-fold cross validation random data split.

**Table 2:** Ground-truth data distribution for missing-reference data split.

Data Type	Positive (pathogenic)	Negative (non-pathogenic)
Training set	2413	2616
Validation set	152	140
Test set	111	817

We trained the GNN model on MetagenomicKG, treating binary pathogen classification as a node classification task, where each Microbe node is identified as either a pathogen or not (see more details in section S3). We then compared it against other sequence-based baseline models using the same training data. Model performance was evaluated based on the six standard metrics: Accuracy (ACC), True Positive Rate (TPR), True Negative Rate (TNR), Area Under the Receiver Operating Characteristic Curve (AUROC), Average Precision (AP), and F1 score (F1) (See more descriptions about these metrics in Section S4). Table 3 presents a performance comparison of the MetagenomicKG -based GNN model and all other baseline models under two different data split strategies. As shown in the table, under the random data split, the baseline models achieved performance comparable to the MetagenomicKG -based GNN model due to high sequence homology between training and test pathogens (i.e. those in the test set). However, under the missing-reference split, where test pathogens lack homologous reference genomes, the performance of sequence-based models declined significantly, particularly in TPR, AP, and F1, indicating their inability to identify unknown pathogens accurately. In contrast, the MetagenomicKG -based GNN model maintained robust performance, achieving approximately a 20 percentage point improvement in TPR over the baseline models, thus demonstrating its advantage in identifying unknown pathogens.

**Table 3:** The performance comparison of pathogen prediction task between GNN with MetagenomicKG and different baseline models based on test dataset using random data split in 10 fold cross validation and missing-reference data split.

Data Split Method	Model	ACC	TPR	TNR	AUROC	AP	F1
10CV Random Split	GNN with MetagenomicKG	**0.966** (0.009)	**0.974** (0.011)	0.960 (0.013)	**0.995** (0.002)	**0.994** (0.002)	0.961 (0.010)
	PaPrBaG	0.901 (0.001)	0.921 (0.001)	0.886 (0.033)	0.968 (0.002)	0.963 (0.002)	0.897 (0.007)
	DeePac*	0.855 (0.029)	0.715 (0.086)	0.959 (0.015)	0.951 (0.008)	0.937 (0.009)	0.805 (0.047)
	RF with 4-mer frequencies	0.962 (0.008)	0.941 (0.002)	0.964 (0.010)	0.988 (0.005)	0.951 (0.005)	**0.962** (0.006)
	SVM with 6-mer embeddings	0.963 (0.009)	0.962 (0.020)	**0.966** (0.011)	0.989 (0.005)	0.989 (0.004)	0.952 (0.011)
Missing-reference Split	GNN with MetagenomicKG	**0.954**	**0.838**	0.970	**0.987**	**0.901**	**0.812**
	PaPrBaG	0.945	0.613	0.990	0.976	0.858	0.727
	DeePac	0.940	0.595	0.987	0.974	0.819	0.702
	RF with 4-mer frequencies	0.938	0.532	**0.993**	0.963	0.810	0.670
	SVM with 6-mer embeddings	0.939	0.550	0.991	0.960	0.803	0.682

1. For 10CV random split, values outside parentheses are averages; values inside are standard deviations.

2. DeePac results use rapid CNN mode for faster training.

These results illustrate that the efficacy of sequence-based models depends on the similarity between features captured from training sequences and those of “unseen” pathogens. However, many pathogenic microbes may exist within the “microbial dark matter” that are dissimilar to known pathogens. In such cases, MetagenomicKG combined with GNN technique proves more effective than the sequence-based models for pathogen predictions.

## 4 Conclusion

We present a novel metagenomics knowledge graph, MetagenomicKG, which integrates widely used taxonomy, functional annotations, pathogenicity information, and other biomedical knowledge from seven relevant databases. This integration aims to enhance the understanding of the complex relationships between microbes and diseases. To the best of our knowledge, MetagenomicKG is the most comprehensive knowledge graph focused on metagenomics to date. To demonstrate its utility, we present three different use cases. First, we show that MetagenomicKG facilitates the exploration of complex relationships among pathogens, AMR-associated proteins, KEGG-based function annotations, diseases, and relevant drugs. Researchers can use our tool to gain new insights and evaluate biological hypotheses. Second, we characterize metagenomic samples by embedding the taxonomic profiles into the topological structure of MetagenomicKG. These embeddings allow for more effective differentiation of microbial communities across samples from different body sites compared to taxonomic profiling alone. Lastly, we demonstrate that combining graph neural network models with MetagenomicKG significantly improves pathogen identification, outperforming sequence-based models, particularly for pathogenic microbes with low sequence similarity to known pathogens.

MetagenomicKG is primarily built upon connections among a collection of biological databases from the Kyoto Encyclopedia of Genes and Genomes (KEGG), which provides comprehensive, high-quality functional and molecular information on biological systems. Using KEGG as the foundation, we further integrate metagenomics-related knowledge including taxonomy, AMR genes, relevant diseases and drugs. The data in MetagenomicKG is updated periodically. In the future, we plan to expand MetagenomicKG by incorporating additional metagenomics-related data sources. As a result, MetagenomicKG can support researchers in uncovering the biological mechanisms underlying microbial impact on human health. Additionally, because all nodes and edges in MetagenomicKG are categorized using the standardized ontologies of Biolink Model, it enables seamless extension and interoperability with other Biolink Model-based knowledge graphs, such as SPOKE (Morris *et al*. 2023), KG-COVID-19 (Reese *et al*. 2021), and BioThings Explorer (Callaghan *et al*. 2023). We believe the applications of MetagenomicKG presented in this work serve as paradigms demonstrating how knowledge graphs can support and advance metagenomic research.

## Supplementary Material

btag421_Supplementary_Data
